# 

**Published:** 1879-09-20

**Authors:** 


### TABLE OF CONTENTS.

ORIGINAL AND SELECTED ARTICLES.

Tobacco Poisoning and its Effects upon the Eye-Sight, by A. W. Calhoun, M.D., 321. Hypodermic Use of Morphia, by B. Greenley, M.D., 325. Is Croupous Pneumonia a Specific Infectious Disease? by S. H. Anderson, M.D., 327. It Steps down from its Airy Throne, by H. W. Carpenter, M.D., 328. Dextro-Quinine, by George Kelly, M.D., 330. Opium Habit Cured by Galvanism, by Wm. F. Hutchinson, M.D., 332. Eczema of the Hand, by John Kent Spender, M.D. 334. Hypodermic Medication in Cholera-Morbus, by John C. Pearson, M.D., 335.

ABSTRACTS AND GLEANINGS.

Value of the Hpyphosphites, 337. Nocturnal Seminal Emissions, 339. Intravenous Injections, 340. Treatment of Unhealthy Local and Syphilitic Sores, by Immersion, 341. Glycerine as a Food, 342. Therapeutics of Singultus, 343. Narcotism from Nutmeg; Nourishment in Typhoid Fever, 344. Kava-Kava; A Novel and Successful Remedy for Hay Fever, 345. Foul Water and Dysentery, 346. Influence of Syphilis on the Cure of Wounds; Transplantation of a Dog's Cornea to the Human Eye, 347. Ethylate of Sodium; Cause of Death in Uterine Injections, 348. Contagium of Syphilis; Honor to Dr. Crawford W. Long; Coffee in Cholera, 349. Chronic Bright's Disease; Poisoning by Rhus Radicans; Contagiousness of Abdominal Typhus; To Hasten the Action of Quinine; Lemon Juice for Hypertrophied Tonsils, 350.

SCIENTIFIC ITEMS.

Sand Blast; Electric Light; Medicines by Galvanism, 351. Fecundity and Sexuality; Sonometry by an Electrical Induction Balance, 352.

PRACTICAL NOTES AND FORMULÆ.

Dyspepsia; Artificial Cataplasm, 353. Topical Use of Ergot; Salicylic Acid; Chaulmoogra Oil, 354. Inhalation of Eucalyptus Oil; To Expel Ascarides; Enuresis in children, 355. Treatment of Gonorrhœa; Modified Dover's Powder; Cardiac Dyspnœa, 356.

EDITORIAL AND MISCELLANEOUS.

Editorial Notices; Why Don't you Write; Book Notices, 357. Receipted; Special Notices, 360.

